# A Novel Evidence Conflict Measurement for Multi-Sensor Data Fusion Based on the Evidence Distance and Evidence Angle

**DOI:** 10.3390/s20020381

**Published:** 2020-01-09

**Authors:** Zhan Deng, Jianyu Wang

**Affiliations:** School of Automation, Nanjing University of Science and Technology, Nanjing 210094, China; wangjyu@njust.edu.cn

**Keywords:** Dempster–Shafer evidence theory, conflict measurement, mutual support degree, Hellinger distance, Pignistic vector angle

## Abstract

As an important method for uncertainty modeling, Dempster–Shafer (DS) evidence theory has been widely used in practical applications. However, the results turned out to be almost counter-intuitive when fusing the different sources of highly conflicting evidence with Dempster’s combination rule. In previous researches, most of them were mainly dependent on the conflict measurement method between the evidence represented by the evidence distance. However, it is inaccurate to characterize the evidence conflict only through the evidence distance. To address this issue, we comprehensively consider the impacts of the evidence distance and evidence angle on conflicts in this paper, and propose a new method based on the mutual support degree between the evidence to characterize the evidence conflict. First, the Hellinger distance measurement method is proposed to measure the distance between the evidence, and the sine value of the Pignistic vector angle is used to characterize the angle between the evidence. The evidence distance indicates the dissimilarity between the evidence, and the evidence angle represents the inconsistency between the evidence. Next, two methods are combined to get a new method for measuring the mutual support degree between the evidence. Afterward, the weight of each evidence is determined by using the mutual support degree between the evidence. Then, the weights of each evidence are utilized to modify the original evidence to achieve the weighted average evidence. Finally, Dempster’s combination rule is used for fusion. Some numerical examples are given to illustrate the effectiveness and reasonability for the proposed method.

## 1. Introduction

In practical applications, most information acquisition is done by sensors. Due to the complexity of the target, the data provided by a single sensor may not be sufficient to obtain all of the information desired for data fusion, providing all the information of target estimation with multiple sensors is, therefore, often required. However, the data derived from multiple sensors could be uncertain or even conflicting. How to deal with uncertain information effectively has been paid much attention. Dempster–Shafer (DS) evidence theory is a powerful tool to represent and deal with uncertain information. It has been widely used in practical problems related to uncertainty modeling and reasoning, such as information fusion [1,2,3,4], fault diagnosis [5,6,7,8,9,10,11], decision-making [12,13,14,15,16], risk assessment [17,18,19,20,21], multi-criteria decision-making [22,23], and pattern recognition [24,25,26,27].

DS evidence theory, also called theories of belief functions, was firstly proposed by Dempster in 1967 [28] and further developed by Shafer in 1976 [29]. DS evidence theory can not only effectively express stochastic uncertainty information, but can also express incomplete and subjective uncertainty information, which can achieve fusion between the evidence without prior information [30]. When using DS evidence theory for fusion, it is necessary to ensure that all sources of evidence to be combined have the same reliability; however, this is difficult to be satisfied in practical applications. If the value of conflict between the evidence provided by the information source is small, Dempster’s combination rule can obtain a good fusion result. However, the anti-intuitive results are often generated when combining the highly conflicting evidence with Dempster’s combination rule, as pointed out by Zadeh in [31]. In order to solve the problem, many researchers have studied this problem and proposed a lot of solutions [32,33,34,35,36,37,38,39,40]. Originally, Shafer used the conflict coefficient *k* to describe the degree of conflict between the evidence [29]. However, the conflict coefficient *k* only expresses the inconsistency between the evidence and cannot accurately reflect the evidence conflict. Murphy proposed to calculate the arithmetic average of *n* evidence, and then used Dempster’s combination rule for fusion [41]. However, Murphy’s method only carried out a simple average, without considering the impacts of conflict between the evidence on the fusion results. In this process, each evidence is assigned the same weight, which seems unreasonable. Jousselme proposed a method to measure the evidence distance in vector space [42], and used the evidence distance to characterize the degree of conflict between the evidence. Martin [43] proposed to use Jousselme distance to evaluate the reliability between the evidence, so as to determine the discount factor of each evidence. According to Jousselme distance, Deng [44] defined the dissimilarity measurement method between the evidence using the dissimilarity between the evidence to represent the evidence conflict. Han [45] used Jousselme distance to measure the similarity between the evidence, and combined information entropy to obtain the credibility of the evidence. Nevertheless, Jousselme distance cannot be enough to fully measure the degree of conflict between the evidence. This is because there are multiple factors affecting the evidence conflicts, while Jousselme distance can only measure one of them.

Smets [46] proposed a Pignistic probabilistic transformation method that approximates the basic probability assignment to a subjective probability measurement. Based on the Pignistic probability transformation function, the Pignistic probability distance between two bodies of evidence was proposed by Liu [47] to characterize the degree of conflict between the evidence. Zhang [48] used the Pignistic probability distance to define the similarity measurement method among the evidence, so as to determine the support degree and weighting factor of each evidence. Chen [49] introduced the Pignistic probability distance to measure the dissimilarity between the evidence, and used the dissimilarity to represent the degree of conflict between the evidence, so as to determine the discounting factor of each evidence. When the body of evidence contain non-singleton focal elements and the basic probability assignment in different evidence have larger differences, the Pignistic probability distance measurement method cannot effectively measure the degree of conflict between the evidence. Xiao [50] proposed a new Belief Jensen–Shannon divergence to measure the dissimilarity and conflict degree between the evidence, so that it determines the credibility of each evidence. A new distance function, which is called supporting probability distance, is proposed by Yu [51] to characterize the degree of conflict among the evidence. Yang [52] used the interval distance of Tran and Duckstein to measure the uncertainty of evidence, and used the uncertainty to characterize the degree of conflict between the evidence. However, these methods only capture one aspect of the evidence conflict. Burger [53] considered that conflict and distance are two different concepts that cannot be interchanged. He explained the relationship between the conflict among evidence and angle from the perspective of geometry. He believed that distance is not the only basic element in geometry, and angle is also important. In DS evidence theory, the essence of Dempster’s combination rule is the orthogonal sum of evidence. Obviously, in Dempster’s opinion, conflict is related to the degree of orthogonality between the evidence. In other words, consistency between the evidence would correspond to collinearity of the mass function, while inconsistency between the evidence would correspond to the orthogonality. In fact, the degree of conflict is related to both evidence distance (measuring dissimilarity between evidence) and evidence angle (measuring inconsistency between evidence). This explains why, for more than two decades now, conflict measurement methods in evidence theory has been regularly challenged by counter-examples. Meanwhile, these counter-examples also show that using a single conflict measurement method cannot accurately measure the degree of conflict between the evidence.

In this paper, we propose a novel method to measure the evidence conflict in vector space, taking into account both the impacts of the evidence distance and evidence angle on conflicts. The evidence distance describes the dissimilarity between the evidence, whereas the evidence angle represents the inconsistency among the evidence. These two measures are mutually complementary in a sense. The Hellinger distance measurement method is proposed to measure the evidence distance, and the sine value of the Pignistic vector angle between the evidence is used to characterize the angle among the evidence. Through combining the evidence distance and evidence angle, a new method to measure the mutual support degree between the evidence is proposed. Afterward, the credibility degree is determined by using the mutual support degree among the evidence, and the weight factor of each evidence is expressed by the credibility degree. The weighting factor of each evidence can be used to modify the original evidence. Based on that, the weighted average evidence can be obtained, and then we use Dempster’s combination rule to combine the weighted average evidence. The rationality and effectiveness of our proposed method are illustrated through numerical examples.

The rest of this paper is organized as follows. We review the basic concepts of evidence theory in Section 2. In Section 3, the Hellinger distance measurement method is proposed to measure the dissimilarity between the evidence. In Section 4, the sine value of the Pignistic vector angle is introduced to characterize the inconsistency among the evidence. A new method to measure the mutual support degree of the evidence is presented in Section 5. The implementation process of our proposed method is described in Section 6. Section 7 illustrates two numerical examples to show the rationality and effectiveness of our proposed method. Section 8 concludes this paper.

## 2. DS Evidence Theory

DS evidence theory is an effective mathematical tool to deal with information fusion. It can not only effectively distinguish “unknown” and “uncertain” information, but also strengthen the complementary relationship between the information and improve the accuracy of decision-making. It has great advantages in the modeling of uncertain information, and represents and combines the uncertain information. In this section, we mainly introduce the basic concepts knowledge of DS evidence theory.

**Definition** **1** **(Frame of discernment).** *Suppose that $\Theta =\{F_{1},\;F_{2},\;\cdots ,\;F_{N}\}$ is a finite nonempty set of N pairs of mutually exclusive and exhaustive hypotheses, the set Θ is called the frame of discernment. The set of all the possible subsets in Θ composed of the power set denoted by $2^{\Theta }$, which include $2^{N}$ elements.*

(1) $$2^{\Theta }=\{\varphi ,\;F_{1},\;F_{2},\;\cdots F_{N},\;\{F_{1},\;F_{2}\}\;,\;\cdots \{F_{1},\;F_{2},\;F_{3}\},\;\cdots \{\Theta \}\}$$

*where φ is an empty set.*

**Definition** **2** **(Basic probability assignment (BPA)).** *Suppose that Θ is a frame of discernment, ∀A⊆Θ, A denotes any subset in the frame of discernment Θ. If the mass function is a mapping m from $2^{\Theta }$ to [0, 1], which satisfies the following conditions:*

(2) $$\{\begin{array}{c} m(\varphi )=0\\ \sum_{A\subseteq \Theta }m(A)=1 \end{array}$$

*m is called as the basic probability assignment. m(A) is the basic probability number of Proposition A. If m(A)>0, A is called the focal element of the basic probability assignment function on Θ. When subset A contains only one element, it is called a single focal element.*

**Definition** **3** **(Dempster’s rule of combination).** *Suppose that the basic probability assignment function of the body of evidence from two independent sources are $m_{1}$ and $m_{2}$, respectively, the corresponding focal elements are $A_{1}\;,\;A_{2}\;,\;\cdots \;,\;A_{k}$ and $B_{1}\;,\;B_{2}\;,\cdots ,\;B_{l}$, respectively, and m is used to represent the new evidence after combining $m_{1}$ and $m_{2}$. Dempster’s rule of combination can be defined as follows:*

(3) $$m(A)=\{\begin{array}{l} 0\;A=\varphi \;\\ \frac{1}{1-k}\sum_{A_{i}\cap B_{j}=A}m_{1}(A_{i})m_{2}(B_{j})\;A\neq \varphi \end{array}$$

(4) $$k=\sum_{A_{i}\cap B_{j}=\varphi }m_{1}(A_{i})m_{2}(B_{j})$$

*where k is the conflict coefficient, which is used to measure the degree of conflict between two bodies of evidence. The larger the conflict between two bodies of evidence is, the larger the value of conflict coefficient k will be. The current research focuses on the fusion of conflicting evidence.*

## 3. Evidence Distance Measure

In the data fusion process, Dempster’s combination rule ignores the conflicting information between the evidence and only emphasizes the consistency between the evidence. When combining the highly conflicting evidence with Dempster’s combination rule, the counter-intuitive result is often obtained. To solve the problem, it is necessary to determine how to quantitatively describe the conflict information. Many researchers put forward to use evidence distance to describe the dissimilarity between the evidence, so as to determine the degree of conflict between the evidence. In this paper, we proposed to use the Hellinger distance to measure the dissimilarity between the evidence.

### 3.1. Hellinger Distance

Distance is a measure that describes the difference between two sets of vectors. The more similar the set of the vectors is, the lower the difference between them. Csiszar [54] and Slivey [55] proposed to use the distance measurement method to measure the dissimilarity between two probability distribution sets in probability theory, which is called the f-divergence method. Hellinger distance is one of the distance measurement methods [56,57].

**Definition** **4** **(Hellinger Distance).** *Hellinger distance is a distance measurement method that is used to measure the dissimilarity between probability distributions, which is defined as follows [58]:*

*Suppose that $P=\{p_{1}\;,p_{2},\;\cdots ,\;p_{n}\}$ and $Q=\{q_{1},\;q_{2},\;\cdots ,\;q_{n}\}$ are two probability distribution vectors of discrete random variable X, and satisfy $p_{i}\geq 0,\;\sum_{i=1}^{n}p_{i}=1;\;q_{i}\geq 0,\;\sum_{i=1}^{n}q_{i}=1\;(i=1,\;2,\;\cdots ,\;n)$, then the Hellinger distance of P to Q is defined as:*

(5) $$Hel(P||Q)=\sqrt{1-\sum_{i=1}^{n}\sqrt{p_{i}q_{i}}}$$

Hellinger distance satisfies the following properties:

(1) Non-negativity: Hel(P||Q)≥0, Hel(P||Q)=0⇔P=Q
(2) Symmetry: Hel(P||Q)=Hel(Q||P)
(3) Triangle inequality: Supposing that $W=\{w_{1},\;w_{2},\;\cdots ,\;w_{n}\}$ is another probability distribution vector of discrete random variable X, then
  (6) Hel(P||Q)≤Hel(P||W)+Hel(W||Q).

It can be seen that the Hellinger distance satisfies the measurable distance conditions. A detailed proof process of the Hellinger distance properties can be found in Ref. [56]. Different from other distance measurement methods, the Hellinger distance is more stable and reliable, and can effectively measure the difference between probability distributions of random variables.

### 3.2. Evidence Dissimilarity Measurement Method Based on the Hellinger Distance

The essence of DS evidence theory is the generalization of probability theory. Hellinger distance is a distance measure that is defined in probability distribution space. So, the above Hellinger distance measurement method can be combined with DS evidence theory, and the Hellinger distance can be used to measure the dissimilarity between the evidence, so as to characterize the degree of conflict between the evidence. In this paper, we define a new dissimilarity measurement method in DS evidence theory. The definition is as follows:

**Definition** **5** **(Hellinger Distance between the evidence).** *Suppose that $\Theta =\{\theta_{1},\;\theta_{2},\;\cdots ,\;\theta_{n}\}$ is a finitely complete set combined with n pairs of mutually exclusive elements. $\theta_{i}$ is a proposition set in the frame of discernment Θ, and $m_{i}$ and $m_{j}$ are two basic probability assignments in the same frame of discernment Θ, so the Hellinger distance between the two bodies of evidence $m_{i}$ and $m_{j}$ is defined as:*

(7) $$Hel(m_{i},m_{j})=\sqrt{1-\sum_{\theta_{i}\in \Theta }\left(\sqrt{m_{i}(\theta_{i})\;m_{j}(\theta_{i})}\right)}$$

*where $\sum_{\theta_{i}\in \Theta }m_{i}(\theta_{i})=1$, $\sum_{\theta_{i}\in \Theta }m_{j}(\theta_{i})=1$.*

Hellinger distance can effectively measure the dissimilarity between the evidence. According to Equation (7), the smaller the Hellinger distance is, the smaller the dissimilarity is, and the less conflicting the evidence is. When two different bodies of evidence are completely in conflict, the Hellinger distance will be almost equal to one. However, when these two bodies of evidence are identical, the Hellinger distance will be close to zero. From the properties of Hellinger distance, the related properties of the Hellinger distance between the evidence can be inferred.

(1) Non-negativity: $0\leq Hel(m_{i},m_{j})\leq 1$
(2) Symmetry: $Hel(m_{i},m_{j})=Hel(m_{j},m_{i})$
(3) Triangle inequality: $Hel(m_{i},m_{j})\leq Hel(m_{i},m_{k})+Hel(m_{k},m_{j})$, where $m_{k}$ is a basic probability assignment in the frame of discernment Θ.

**Proof.**  Since $m_{i}$ and $m_{j}$ are basic probability assignments in the frame of discernment Θ, Equation (7) can be transformed into the following form:

(8) $$Hel(m_{i},m_{j})=\sqrt{\frac{1}{2}\sum_{\theta_{s}\in \Theta }{\left(\sqrt{m_{i}(\theta_{s})}-\sqrt{m_{j}(\theta_{s})}\right)}^{2}}$$

According to Equation (8), it can be seen that non-negativity property and symmetry property are satisfied by the requirements. The triangle inequality of Equation (6) is proved as follows:

According to Equation (8), we can get:

$$\begin{array}{c} Hel(m_{i},m_{j})=\sqrt{\frac{1}{2}\sum_{\theta_{s}\in \Theta }{\left(\sqrt{m_{i}(\theta_{s})}-\sqrt{m_{j}(\theta_{s})}\right)}^{2}}\\ Hel(m_{i},m_{k})=\sqrt{\frac{1}{2}\sum_{\theta_{s}\in \Theta }{\left(\sqrt{m_{i}(\theta_{s})}-\sqrt{m_{k}(\theta_{s})}\right)}^{2}}\\ Hel(m_{k},m_{j})=\sqrt{\frac{1}{2}\sum_{\theta_{s}\in \Theta }{\left(\sqrt{m_{k}(\theta_{s})}-\sqrt{m_{j}(\theta_{s})}\right)}^{2}} \end{array}$$

Because $0\leq Hel(m_{i},m_{j})\leq 1$

Therefore

$$\begin{array}{c} Hel(m_{i},m_{j})\leq Hel(m_{i},m_{k})+Hel(m_{k},m_{j})\\ \Leftrightarrow Hel{\left(m_{i},m_{j}\right)}^{2}\leq {\left(Hel(m_{i},m_{k})+Hel(m_{k,}m_{j})\right)}^{2} \end{array}$$

That is

(9) $${\left(\sqrt{\frac{1}{2}\sum_{\theta_{s}\in \Theta }{\left(\sqrt{m_{i}(\theta_{s})}-\sqrt{m_{k}(\theta_{s})}\right)}^{2}}+\sqrt{\frac{1}{2}\sum_{\theta_{s}\in \Theta }{\left(\sqrt{m_{k}(\theta_{s})}-\sqrt{m_{j}(\theta_{s})}\right)}^{2}}\right)}^{2}\geq {\left(\sqrt{\frac{1}{2}\sum_{\theta_{s}\in \Theta }{\left(\sqrt{m_{i}(\theta_{s})}-\sqrt{m_{j}(\theta_{s})}\right)}^{2}}\right)}^{2}$$

That proof

(10) $$\frac{1}{2}\sum_{\theta_{s}\in \Theta }{\left(\sqrt{m_{i}(\theta_{s})}-\sqrt{m_{k}(\theta_{s})}\right)}^{2}+\frac{1}{2}\sum_{\theta_{s}\in \Theta }{\left(\sqrt{m_{k}(\theta_{s})}-\sqrt{m_{j}(\theta_{s})}\right)}^{2}\geq \frac{1}{2}\sum_{\theta_{s}\in \Theta }{\left(\sqrt{m_{i}(\theta_{s})}-\sqrt{m_{j}(\theta_{s})}\right)}^{2}$$

Only if Inequality (10) is held, Inequality (9) is necessary to be held. The problem is transformed into proving that Inequality (10) is held.

Simplify Inequality (10) can be obtained:

(11) $$\begin{array}{c} \frac{1}{2}\sum_{\theta_{s}\in \Theta }{\left(\sqrt{m_{i}(\theta_{s})}-\sqrt{m_{k}(\theta_{s})}\right)}^{2}+\frac{1}{2}\sum_{\theta_{s}\in \Theta }{\left(\sqrt{m_{k}(\theta_{s})}-\sqrt{m_{j}(\theta_{s})}\right)}^{2}-\frac{1}{2}\sum_{\theta_{s}\in \Theta }{\left(\sqrt{m_{i}(\theta_{s})}-\sqrt{m_{j}(\theta_{s})}\right)}^{2}\\ =\sum_{\theta_{s}\in \Theta }\left(\sqrt{m_{k}(\theta_{s})}-\sqrt{m_{i}(\theta_{s})}\right)\left(\sqrt{m_{k}(\theta_{s})}-\sqrt{m_{j}(\theta_{s})}\right) \end{array}$$

Taking the logarithm, it can be obtained that:

$$=\sum_{\theta_{s}\in \Theta }\left(\ln\left(\frac{e^{\sqrt{m_{k}(\theta_{s})}}}{e^{\sqrt{m_{i}(\theta_{s})}}}\right)+\ln\left(\frac{e^{\sqrt{m_{k}(\theta_{s})}}}{e^{\sqrt{m_{j}(\theta_{s})}}}\right)\right)$$

In this section, we need to use the information entropy inequality as follows:

(12) $$\sum_{i=1}^{n}w_{i}\ln w_{i}\geq \sum_{i=1}^{n}w_{i}\ln p_{i}$$

According to the information entropy inequality, we can obtain that

$$\sum_{\theta_{s}\in \Theta }e^{\sqrt{m_{k}(\theta_{s})}}\ln\left(\frac{e^{\sqrt{m_{k}(\theta_{s})}}}{e^{\sqrt{m_{i}(\theta_{s})}}}\right)\geq 0,\;\sum_{\theta_{s}\in \Theta }e^{\sqrt{m_{k}(\theta_{s})}}\ln\left(\frac{e^{\sqrt{m_{k}(\theta_{s})}}}{e^{\sqrt{m_{j}(\theta_{s})}}}\right)\geq 0$$

So

$$\sum_{\theta_{s}\in \Theta }\ln\left(\frac{e^{\sqrt{m_{k}(\theta_{s})}}}{e^{\sqrt{m_{i}(\theta_{s})}}}\right)\geq 0,\;\sum_{\theta_{s}\in \Theta }\ln\left(\frac{e^{\sqrt{m_{k}(\theta_{s})}}}{e^{\sqrt{m_{j}(\theta_{s})}}}\right)\geq 0$$

Therefore

$$\sum_{\theta_{s}\in \Theta }\left(\ln\left(\frac{e^{\sqrt{m_{k}(\theta_{s})}}}{e^{\sqrt{m_{i}(\theta_{s})}}}\right)+\ln\left(\frac{e^{\sqrt{m_{k}(\theta_{s})}}}{e^{\sqrt{m_{j}(\theta_{s})}}}\right)\right)\geq 0$$

That is

$$\frac{1}{2}\sum_{\theta_{s}\in \Theta }{\left(\sqrt{m_{i}(\theta_{s})}-\sqrt{m_{k}(\theta_{s})}\right)}^{2}+\frac{1}{2}\sum_{\theta_{s}\in \Theta }{\left(\sqrt{m_{k}(\theta_{s})}-\sqrt{m_{j}(\theta_{s})}\right)}^{2}-\frac{1}{2}\;\sum_{\theta_{s}\in \Theta }{\left(\sqrt{m_{i}(\theta_{s})}-\sqrt{m_{j}(\theta_{s})}\right)}^{2}\geq 0$$

Therefore, Inequality (9) is held. The triangle inequality of the Hellinger distance is held.

A few simple examples are given to illustrate the related properties and specific calculation processes of the Hellinger distance between the evidence. □

**Example** **1.** *Suppose that there are two independent bodies of evidence $m_{1}$ and $m_{2}$ in the frame of discernment Θ={A, B, C, D}, and their basic probability assignments are described as follows:*

$$\begin{array}{l} m_{1}:\;m_{1}(A)=0.25\;m_{1}(B)=0.25\;m_{1}(C)=0.25\;m_{1}(D)=0.25\\ m_{2}:\;m_{2}(A)=0.25\;m_{2}(B)=0.25\;m_{2}(C)=0.25\;m_{2}(D)=0.25 \end{array}$$

As can be seen from Example 1, two bodies of evidence $m_{1}$ and $m_{2}$ have the same basic probability assignment, and they are two identical bodies of evidence. The specific calculation process of the Hellinger distance between two bodies of evidence $m_{1}$ and $m_{2}$ is as follows:

$$Hel(m_{1},m_{2})=\sqrt{1-\left(\sqrt{0.25\times 0.25}+\sqrt{0.25\times 0.25}+\sqrt{0.25\times 0.25}+\sqrt{0.25\times 0.25}\right)}=0$$

According to the results, when two bodies of evidence have the same basic probability assignment, the Hellinger distance between two bodies of evidence is zero. These two bodies of evidence are similar, which is consistent with the intuitive analysis results.

**Example** **2.** *Suppose that there are two independent bodies of evidence $m_{1}$ and $m_{2}$ in the frame of discernment Θ={a,b,c}, and their basic probability assignments are described as follows:*

$$\begin{array}{c} m_{1}:\;m_{1}(a)=0.5\;m_{1}(b)=0.3\;m_{1}(c)=0.1\;m_{1}(\Theta )=0.1\\ m_{2}:\;m_{2}(a)=0.3\;m_{2}(b)=0.5\;m_{2}(c)=0\;m_{2}(\Theta )=0.2 \end{array}$$

The specific calculation process of the Hellinger distance between the two bodies of evidence $m_{1}$ and $m_{2}$ is as follows:

$$Hel(m_{1},m_{2})=\sqrt{1-\left(\sqrt{0.5\times 0.3}+\sqrt{0.3\times 0.5}+\sqrt{0.1\times 0}+\sqrt{0.1\times 0.2}\right)}=0.2898$$

**Example** **3.** *Suppose that there are three independent bodies of evidence $m_{1}$, $m_{2}$, and $m_{3}$ in the frame of discernment $\Theta =\{\theta_{1},\;\theta_{2}\}$, and their basic probability assignments are described as follows:*

$$\begin{array}{c} m_{1}:\;m_{1}(\theta_{1})=0.99\;m_{1}(\theta_{2})=0.01\\ m_{2}:\;m_{2}(\theta_{1})=0.90\;m_{2}(\theta_{2})=0.10\\ m_{3}:\;m_{3}(\theta_{1})=0.01\;m_{3}(\theta_{2})=0.99 \end{array}$$

It can be seen from Example 3 that the two bodies of evidence $m_{1}$ and $m_{2}$ assign most of the belief values to proposition $\theta_{1}$, while the body of evidence $m_{3}$ assigns most of the belief values to proposition $\theta_{2}$. The evidence $m_{3}$ is in high conflict with the other two bodies of evidence. The Hellinger distance between any two pairs of the three bodies of evidence is calculated, respectively. The specific calculation process is as follows:

$$\begin{array}{c} Hel(m_{1},m_{2})=\sqrt{1-\left(\sqrt{0.99\times 0.9}+\sqrt{0.01\times 0.10}\right)}=0.1563\\ Hel(m_{1},m_{3})=\sqrt{1-\left(\sqrt{0.99\times 0.01}+\sqrt{0.01\times 0.99}\right)}=0.8950\\ Hel(m_{2},m_{3})=\sqrt{1-\left(\sqrt{0.90\times 0.01}+\sqrt{0.10\times 0.99}\right)}=0.7684 \end{array}$$

It can be noticed that $Hel(m_{1},m_{2})+Hel(m_{2},m_{3})=0.9274$ is greater than $Hel(m_{1},m_{3})=0.8950$, so $Hel(m_{1},m_{2})+Hel(m_{2},m_{3})>Hel(m_{1},m_{3})$ satisfies the triangle inequality property of the Hellinger distance between the evidence. The Hellinger distance between the two bodies of evidence $m_{1}$ and $m_{2}$ is smaller than that between two bodies of evidence $m_{1}$ and $m_{3}$, and it indicates that the dissimilarity between the two bodies of evidence $m_{1}$ and $m_{2}$ is less than that between the two bodies of evidence $m_{1}$ and $m_{3}$, which is consistent with the intuitive analysis.

**Example** **4.** *Suppose that there are two independent bodies of evidence $m_{1}$ and $m_{2}$ in the frame of discernment Θ={A,B}, and their basic probability assignments are described as follows:*

$$\begin{array}{c} m_{1}:\;m_{1}(A)=1\;m_{1}(B)=0\\ m_{2}:m_{2}(A)=x\;m_{2}(B)=1-x \end{array}$$

**Figure 1:**
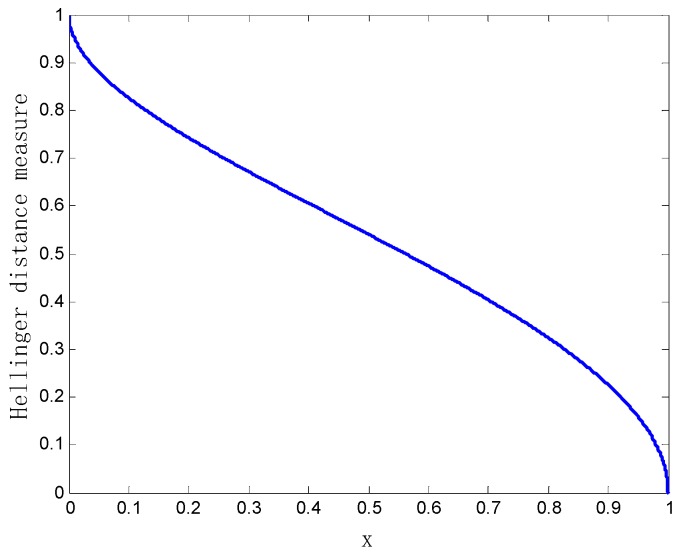
The variation curve of the Hellinger distance between the evidence.

When the parameter x changes in the range of [0, 1], the variation of the Hellinger distance between the two bodies of evidence $m_{1}$ and $m_{2}$ is shown in Figure 1.

As can be seen from Figure 1, when x tends to one, the Hellinger distance between the two bodies of evidence $m_{1}$ and $m_{2}$ gradually goes to zero. This indicates that the two bodies of evidence $m_{1}$ and $m_{2}$ are almost the same. Conversely, when x approaches zero, the Hellinger distance between the two bodies of evidence $m_{1}$ and $m_{2}$ gradually approaches one, and the bodies of evidence $m_{1}$ and $m_{2}$ are completely in conflict. In a word, the bounded property of the Hellinger distance measurement method [0, 1] is verified in this example.

**Example** **5.** *Suppose that there are three independent bodies of evidence $m_{1}$, $m_{2}$, and $m_{3}$ in the frame of discernment Θ={a,b,c}, and their basic probability assignments are described as follows:*

$$\begin{array}{l} m_{1}:\;m_{1}(a)=0.5\;m_{1}(b)=0.3\;m_{1}(c)=0.2\\ m_{2}:\;m_{2}(a)=0.2\;m_{1}(b)=0.3\;m_{1}(c)=0.5\\ m_{3}:\;m_{3}(a)=0.0\;m_{1}(b)=0.1\;m_{1}(c)=0.9 \end{array}$$

As is shown in Example 5, we can see that the two bodies of evidence $m_{2}$ and $m_{3}$ have great belief values to support the proposition c, while $m_{1}$ has a great belief value to support the proposition a. Therefore, it can be concluded that the degree of dissimilarity between $m_{1}$ and $m_{2}$ is larger than that between $m_{2}$ and $m_{3}$. The Hellinger distance between the two bodies of evidence $m_{1}$ and $m_{2}$ is $Hel(m_{1},m_{2})=0.2598$, and for the two bodies of evidence $m_{2}$ and $m_{3}$, the Hellinger distance is $Hel(m_{2},m_{3})=0.3950$. That is, the degree of dissimilarity between two bodies of evidence $m_{2}$ and $m_{3}$ is larger than that between the two bodies of evidence $m_{1}$ and $m_{2}$, which is inconsistent with the intuitive analysis. This simple example shows that only using the distance measurement is not good enough to properly characterize the degree of conflict between the evidence. Therefore, we consider combining the use of another method to describe more efficiently the degree of conflict between the evidence. This new method will be a complementary measurement for the distance measurement and help to define a new efficient conflict measurement method.

## 4. The Evidence Angle Measurement Method

In Example 5, we note that mere use of the evidence distance cannot accurately measure the degree of conflict between the evidence. In the evidence conflict measurement study, the idea of defining conflict measure as a distance was proposed by many researchers. Burger [53] considered that conflict and distance are two different concepts that cannot be interchanged. Burger explained the relationship between the conflict among evidence and angle from the perspective of geometry. So, we use the evidence angle to describe the degree of conflict between the evidence.

In this section, we introduce the Pignistic vector angle measure to describe the evidence conflict. Zhao [59], from the perspective of vector space, regarded each body of evidence as a spatial vector, combining the concept of Pignistic probability transformation and vector angle. Based on that, he proposed to measure the degree of inconsistency between the evidence by using the sine value of the Pignistic vector angle between the evidence, and then used the inconsistency to characterize the degree of conflict between the evidence. This method is defined as follows:

For a frame of discernment Θ that contains *n* elements, there are $2^{n}$ possible hypotheses in the problem domain. In order to correspond to the dimension of space vector, we should transform the belief assignment vector into an *n*-dimensional vector, where the converted *n*-dimensional vector is obtained from the Pignistic probability function introduced in Definition 6.

**Definition** **6** **(Pignistic probability transformation function).** *Suppose that m(A) is a basic probability assignment in the frame of discernment Θ, then the Pignistic probability transformation function $BetP_{m}$ is defined as follows:*

(13) $$BetP_{m}(A)=\sum_{\forall A\subseteq \Theta ,B\subseteq \Theta }\frac{\left|A\cap B\right|}{\left|B\right|}\frac{m(B)}{1-m(\varphi )}$$

*where m(φ)≠1, |A| is the number of elements in set A.*

**Definition** **7** **(Measure the angle between the evidence).** *In the frame of discernment Θ, there are the two bodies of evidence $m_{i}$ and $m_{j}$. The Pignistic probability transforms of the two bodies of evidence $m_{i}$ and $m_{j}$ are $BetP_{m_{1}}$ and $BetP_{m2}$ respectively, then the sine value of Pignistic vector angle between the two bodies of evidence is defined as follows:*

(14) $$Sin(m_{i},\;m_{j})=\sqrt{1-{\left(\frac{BetP_{m_{i}}\cdot BetP_{m_{j}}}{\left\|BetP_{mi}\right\|\cdot \left\|BetP_{mj}\right\|}\right)}^{2}}$$

The evidence from the conflict measurement function of the Pignistic vector angle can effectively measure the inconsistency between the evidence. According to Equation (14), the larger the sine value between the evidence is, the greater the inconsistency between the evidence is. If two bodies of evidence are completely in conflict, the sine value between the wo bodies of evidence is equal to one. Some simple examples are used to illustrate the calculation process of the sine value of the Pignistic vector angle between the evidence is as follows:

**Example** **6.** *Suppose that there are two independent bodies of evidence $m_{1}$ and $m_{2}$ in the frame of discernment $\Theta =\{\theta_{1},\;\theta_{2},\;\theta_{3}\}$, and their basic probability assignments are described as follows:*

$$\begin{array}{c} m_{1}:\;m_{1}(\theta_{1})=0.5\;m_{1}(\theta_{2})=0.1\;m_{1}(\theta_{3})=0.2\;m_{1}(\theta_{2},\;\theta_{3})=0.2\\ m_{2}:\;m_{2}(\theta_{1})=0.5\;m_{2}(\theta_{2})=0.1\;m_{2}(\theta_{3})=0.2\;m_{2}(\theta_{2},\;\theta_{3})=0.2 \end{array}$$

As can be seen from Example 6, the two bodies of evidence $m_{1}$ and $m_{2}$ have the same basic probability assignment, and they are two identical bodies of evidence. The specific calculation process for the sine value of the Pignistic vector angle between $m_{1}$ and $m_{2}$ is as follows:

First, the Pignistic probability transformation was performed for each body of evidence.

$$\begin{array}{c} m_{1}:\;BetP_{m_{1}}(\theta_{1})=0.5\;BetP_{m_{1}}(\theta_{2})=0.2\;BetP_{m_{1}}(\theta_{3})=0.3\\ m_{2}:\;BetP_{m_{2}}(\theta_{1})=0.5\;BetP_{m_{2}}(\theta_{2})=0.2\;BetP_{m_{2}}(\theta_{3})=0.3\\ \left\|BetP_{m_{1}}\right\|=\left\|BetP_{m_{2}}\right\|=0.6164\\ Sin(m_{1},m_{2})=\sqrt{1-{\left(\frac{0.5\times 0.5+0.2\times 0.2+0.3\times 0.3}{0.6164\times 0.6164}\right)}^{2}}=0 \end{array}$$

As can be seen from the results, when two bodies of evidence are the same, the sine value of the Pignistic vector angle between the evidence is zero, which the two bodies of evidence are consistent.

**Example** **7.** *Suppose that there are two independent bodies of evidence $m_{1}$ and $m_{2}$ in the frame of discernment Θ={A,B}, and their basic probability assignments are described as follows:*

$$\begin{array}{c} m_{1}:m_{1}(A)=0.5\;m_{1}(B)=0.5\\ m_{2}:m_{2}(A)=x\;m_{2}(B)=1-x \end{array}$$

**Figure 2:**
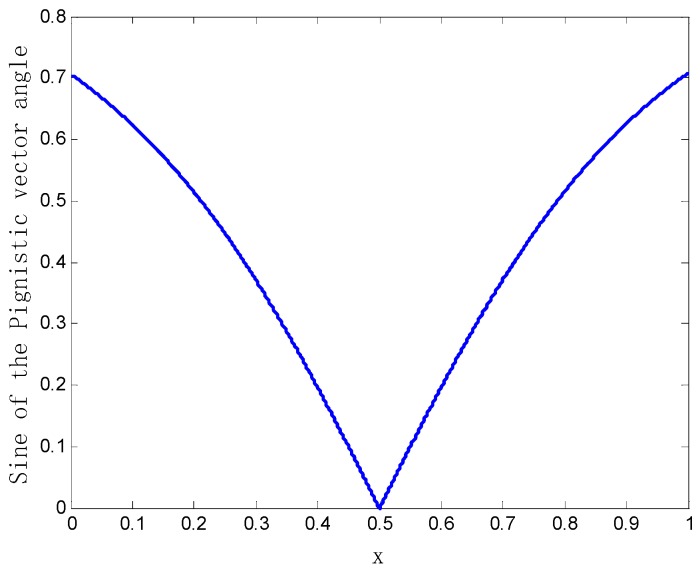
The variation curve of the sine value of Pignistic vector angle between the evidence.

*When the parameter x changes in the range of [0, 1], the variation of the sine value of the Pignistic vector angle between the two bodies of evidence $m_{1}$ and $m_{2}$ is shown in Figure 2.*

As shown in Figure 2, when parameter x approaches 0.5, the sine value of the Pignistic vector angle between $m_{1}$ and $m_{2}$ approaches zero, and $m_{1}$ and $m_{2}$ are almost the same. Conversely, when parameter x approaches zero or one, the sine value of the Pignistic vector angle between the two bodies of evidence $m_{1}$ and $m_{2}$ approaches 0.7071. This explains the phenomenon intuitively where the two bodies of evidence $m_{1}$ and $m_{2}$ are completely in conflict.

**Example** **8.** *Suppose there are three independent bodies of evidence $m_{1}$, $m_{2}$, and $m_{3}$ in the frame of discernment $\Theta =\{\theta_{1},\theta_{2},\theta_{3}\}$, and their basic probability assignments are described as follows:*

$$\begin{array}{c} m_{1}:\;m_{1}(\theta_{1})=1/6\;m_{1}(\{\theta_{2},\theta_{3}\})=1/3\;m_{1}(\Theta )=1/2\\ m_{2}:\;m_{2}(\theta_{1})=1/3\;m_{2}(\theta_{2})=1/3\;m_{2}(\theta_{3})=1/3\\ m_{3}:\;m_{3}(\Theta )=1 \end{array}$$

As shown in Example 8, we can see that there exists conflict among the three bodies of evidence. Therefore, the degree of inconsistency between the evidence is not 0. The sine value of the Pignistic vector angle between two bodies of evidence $m_{1}$ and $m_{2}$, and between the two bodies of evidence $m_{1}$ and $m_{3}$, can be calculated as follows:

$$Sin(m_{1},m_{2})=0\;Sin(m_{1},m_{3})=0$$

From the results, it can be seen that the degree of inconsistency between the evidence is 0, which contradicts the intuitive analysis. Note that, in some cases, the Pignistic vector angle cannot effectively measure the conflict between the evidence.

In this example, when we use the Hellinger distance to measure the dissimilarity between the evidence, the following results can be achieved.

$$Hel(m_{1},m_{2})=0.8742\;Hel(m_{1},m_{3})=0.5412$$

It can be seen that the degree of dissimilarity between the evidence is not 0. This shows that there is a certain conflict between the evidence, which is consistent with the intuitive analysis.

This example shows that only use the sine value of the Pignistic vector angle between the evidence cannot effectively measure the degree of conflict between the evidence. It is necessary to combine another criterion to jointly describe the conflict degree between the evidence. In fact, the conflict measurement method based on evidence distance and evidence vector angle is complementary. They separately capture the different aspects of conflict between the evidence. If the conflict among the evidence is only characterized by using the dissimilarity between the evidence, it cannot reflect the impact of the inconsistency between the evidence on the conflict. On the contrary, if only use the inconsistency between the evidence to represent the conflict, the effect of the dissimilarity between the evidence on the conflict is not taken into account. So, considering the impact of the dissimilarity and inconsistency between the evidence on conflict, we propose to comprehensively measure the mutual support degree between the evidence by using the evidence distance and evidence angle. After that, we use the mutual support degree of the evidence to characterize the degree of conflict between the evidence.

## 5. Support Degree Measurement between the Evidence

In DS evidence theory, the essence of Dempster’s combination rule is the orthogonal sum of evidence. Obviously, in Dempster’s mind, the conflict is related to the degree of orthogonality between the evidence. In other words, the consistency between the evidence would correspond to collinearity of the belief function, while the inconsistency between the evidence would correspond to orthogonality. It follows that the degree of conflict is related to both evidence distance (measuring dissimilarity between evidence) and evidence angle (measuring inconsistency between evidence). The evidence distance and evidence angle are enough to describe the degree of conflict between the evidence. Synthesizing the evidence distance and evidence angle, we propose to use the mutual support degree between the evidence to characterize the degree of conflict between the evidence in order to capture the two main aspects that affect the evidence conflicts. The mutual support degree between the evidence is defined by fusing the dissimilarity and inconsistency between the evidence.

**Definition** **8** **(Mutual support degree measure between the evidence).** *In the frame of discernment Θ, there are two bodies of evidence $m_{i}$ and $m_{j}$. $Hel(m_{i},m_{j})$ is the dissimilarity measure between the two bodies of evidence $m_{i}$ and $m_{j}$, and $Sin(m_{i},m_{j})$ is the measure of the inconsistency between the two bodies of evidence $m_{i}$ and $m_{j}$. The mutual support degree between the two bodies of evidence $m_{i}$ and $m_{j}$ is defined as follows:*

(15) $$Sup(m_{i},m_{j})=(1-Hel(m_{i},m_{j}))(1-Sin(m_{i},m_{j}))$$

The proposed method satisfies the two following important properties:

1. Non-negativity: $0\leq Sup(m_{i},m_{j})\leq 1$
2. Symmetry: $Sup(m_{i},m_{j})=Sup(m_{j},m_{i})$

The larger the mutual support degree between the evidence is, the less conflict between the evidence is. When two bodies of evidence are completely in conflict, the mutual support degree between the evidence is close to zero. In this paper, we use the mutual support degree between the evidence to determine the weighting factor of each body of evidence.

The following examples are given to illustrate the effectiveness of the proposed method for conflict measurement. In Example 5, we only use the dissimilarity measurement method to characterize the evidence conflict, while the results are inconsistent with the intuitive analysis. The proposed measurement method is used to calculate the mutual support degree between the evidence in Example 5, which is shown as follows.

First, the dissimilarity and inconsistency between the two bodies of evidence $m_{1}$ and $m_{2}$, and between the two bodies of evidence $m_{2}$ and $m_{3}$, are calculated, respectively, and the results are as follows:

$$\begin{array}{c} Hel(m_{1},m_{2})=0.2598\;Hel(m_{2},m_{3})=0.3950\\ Sin(m_{1},m_{2})=0.6461\;Sin(m_{2},m_{3})=0.5103 \end{array}$$

According to Equation (15), we can achieve the mutual support degree between the two bodies of evidence $m_{1}$ and $m_{2}$, and between two bodies of evidence $m_{2}$ and $m_{3}$, respectively:

$$Sup(m_{1},m_{2})=0.2620\;Sup(m_{2},m_{3})=0.2963$$

The mutual support degree between $m_{2}$ and $m_{3}$ is larger than that between $m_{1}$ and $m_{2}$ based on the results; that is, the degree of conflict between $m_{2}$ and $m_{3}$ is smaller than that between $m_{1}$ and $m_{2}$, which is consistent with the intuitive analysis. This simple example shows that the proposed method can solve the problem existing in a single measurement method, and can accurately measure the degree of conflict between the evidence. It can be noted that, in Example 5, the dissimilarity between the evidence cannot effectively measure the conflict between the evidence; however, the inconsistency measurement between the evidence can be. This is due to the fact that the dissimilarity measurement and inconsistency measurement between the evidence capture the different aspects of the conflict, respectively.

In Example 8, we only use the inconsistency measurement method to describe the evidence conflict, while the results are inconsistent with the intuitive analysis. The proposed method is used to calculate the mutual support degree between the evidence in Example 8, which is shown as follows.

First, the dissimilarity and inconsistency between the two bodies of evidence $m_{1}$ and $m_{2}$, and between the two bodies of evidence $m_{1}$ and $m_{3}$ are calculated, respectively, and the results are as follows:

$$\begin{array}{c} Hel(m_{1},m_{2})=0.8742\;Hel(m_{1},m_{3})=0.5412\\ Sin(m_{1},m_{2})=0\;Sin(m_{1},m_{3})=0 \end{array}$$

According to Equation (15), the mutual support degree between the two bodies of evidence $m_{1}$ and $m_{2}$, and between the two bodies of evidence $m_{1}$ and $m_{3}$ can be obtained, respectively, as follows:

$$Sup(m_{1},m_{2})=0.1258\;Sup(m_{1},m_{3})=0.4588$$

The mutual support degree between the evidence is different. This example further illustrates the effectiveness of our proposed method.

**Example** **9.** *Suppose there are two independent bodies of evidence $m_{1}$ and $m_{2}$ in the frame of discernment Θ={A,B}, and their basic probability assignments are described as follows:*

$$\begin{array}{c} m_{1}:\;m_{1}(A)=1\;m_{1}(B)=0\\ m_{2}:m_{2}(A)=x\;m_{2}(B)=1-x \end{array}$$

**Figure 3:**
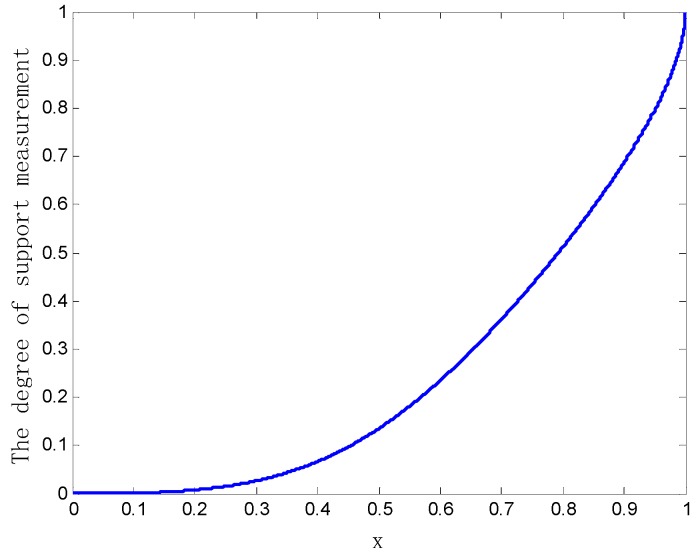
The variation curve of the mutual support degree between the evidence.

When the parameter x changes in the range of [0, 1], the variation of the mutual support degree between the two bodies of evidence $m_{1}$ and $m_{2}$ is shown in Figure 3.

As can be seen from Figure 3, when x tends to 1, the two bodies of evidence $m_{1}$ and $m_{2}$ are almost the same, and the mutual support degree between them gradually goes to 1. Conversely, when x is close to 0, the two bodies of evidence $m_{1}$ and $m_{2}$ are completely in conflict, and the mutual support degree between them gradually approaches 0. In short, the bounded property of the mutual support degree measurement method between the evidence [0, 1] is verified in this example.

Several above examples show that our proposed method takes into account the different aspects that affect the evidence conflict, and can accurately measure the degree of conflict between the evidence.

## 6. Determining the Weighting Factors of the Body of Evidence

In the process of multi-sensor data fusion, due to the different information collection ability and accuracy of different sensors, the evidence provided by sensors is not all of the same reliability. In fact, the evidence with a high degree of conflict will lead to unreasonable fusion results. Therefore, we need to determine the credibility of each body of evidence before fusion. In this paper, we use the mutual support degree between the evidence to characterize the credibility of each evidence. Firstly, the dissimilarity and inconsistency between the evidence are measured by using the Hellinger distance and the sine value of the Pignistic vector angle, and then the mutual support degree between the evidence is obtained based on the dissimilarity and inconsistency between the evidence. When the evidence is more highly supported by another evidence, the more credible the evidence is. Secondly, the mutual support degree between the evidence is used to measure the credibility of each evidence. After that, the credibility degree of the evidence is used as the weighting factor for the evidence, and the original evidence is modified by using the weighting factor to obtain the weighted average evidence. Finally, we use Dempster’s combination rule to combine the weighted average evidence. The implementation process of the proposed method is shown as below.

**Step 1:** According to Equations (7) and (14), the dissimilarity and inconsistency between the two bodies of evidence $m_{i}$(i=1, 2,⋯, n) and $m_{j}$(j=1, 2, ⋯,n) can be obtained, which are expressed as $Hel(m_{i},m_{j})$ and $Sin(m_{i},m_{j})$.

**Step 2:** According to the dissimilarity and inconsistency between the two bodies of evidence $m_{i}$ and $m_{j}$, the mutual support degree between the two bodies of evidence $m_{i}$ and $m_{j}$ is calculated by using Equation (15), and is denoted as $Sup_{ij}$. The mutual support degree measurement matrix among the evidence can be constructed as follows:

(16) $$SM=\left[\begin{array}{cccc} 1 & Sup_{12} & \cdots & Sup_{1n}\\ Sup_{21} & 1 & \cdots & Sup_{2n}\\ \vdots & \vdots & \vdots & \vdots \\ Sup_{n1} & Sup_{n2} & \cdots & Sup_{nn} \end{array}\right]$$

**Step 3:** The average support degree of the body of evidence $m_{i}$ can be calculated by using Equation (17) as follows:

(17) $$Sup(m_{i})=\frac{\sum_{j=1,j\neq i}^{n}Sup_{ij}}{n-1}\;1\leq i\leq n\;;\;1\leq j\leq n$$

**Step 4:** The credibility degree of the body of evidence $m_{i}$ is defined as follows:

(18) $$Cred(m_{i})=\frac{Sup(m_{i})}{\sum_{i=1}^{n}Sup(m_{i})}\;1\leq i\leq n$$

**Step 5:** The greater the credibility of the evidence is, the more important the evidence is. Therefore, the credibility degree of the body of evidence $m_{i}$ is considered as the weighting factor in terms of evidence $m_{i}$.

(19) $$\omega_{i}=Cred(m_{i})$$

**Step 6:** On the basis of the weighting factor of the evidence $m_{i}$, the weighted average evidence can be obtained as follows:

(20) $$WAE(m)=\sum_{i=1}^{n}\left(\omega_{i}\times m_{i}\right)\;1\leq i\leq n$$

**Step 7:** The weighted average evidence is fused *n* − 1 times through Dempster’s combination rule, and then the final combination result can be obtained.

**Figure 4:**
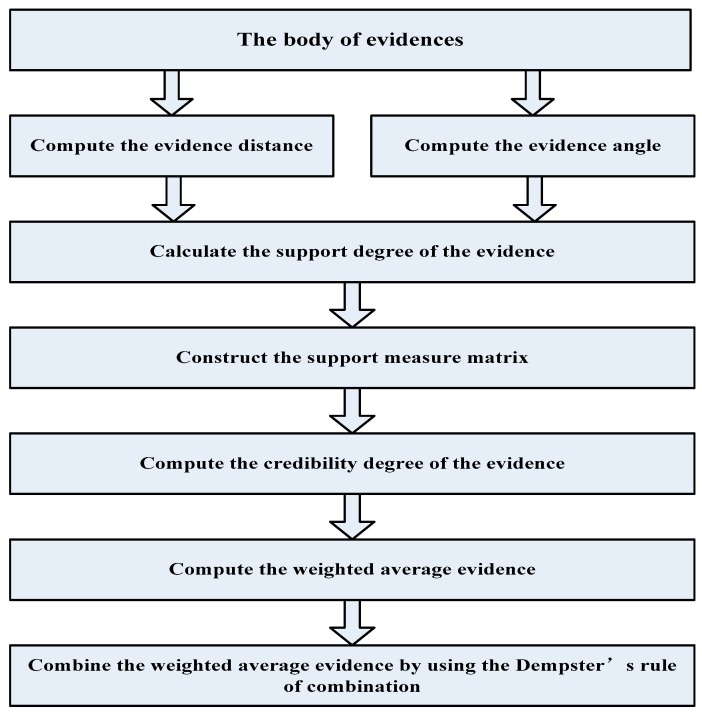
The flowchart of our proposed method.

The flowchart of our proposed method is shown in Figure 4.

The following examples illustrate the implementation process of our proposed method and its advantages.

**Example** **10.** *Suppose that there are two independent bodies of evidence $m_{1}$ and $m_{2}$ in the frame of discernment $\Theta =\{\theta_{1},\;\theta_{2},\;\theta_{3}\}$, and their basic probability assignments are described as follows:*

$$\begin{array}{c} m_{1}:\;m_{1}(\theta_{1})=0.99\;m_{1}(\theta_{2})=0\;m_{1}(\theta_{3})=0.01\\ m_{2}:\;m_{2}(\theta_{1})=0\;m_{2}(\theta_{2})=0.90\;m_{1}(\theta_{3})=0.10 \end{array}$$

As can be seen in Example 10, the evidence $m_{1}$ strongly supports the proposition $\theta_{1}$, whereas $m_{2}$ strongly supports the proposition $\theta_{2}$. There is a high degree of conflict between the two bodies of evidence $m_{1}$ and $m_{2}$. From the symmetry of the mutual support degree, the mutual support degree of the evidence $m_{1}$ to evidence $m_{2}$ is equal to that of the evidence from $m_{2}$ to evidence $m_{1}$. Therefore, the two bodies of evidence have the same credibility, and the weight factor of both bodies of evidence is 0.5. According to the weight factor of each evidence, the weighted average evidence is calculated as follows:

$$m(\theta_{1})=0.495\;m_{2}(\theta_{2})=0.45\;m(\theta_{3})=0.055$$

Finally, the weighted average evidence is fused by using Dempster’s combination rule, and the fusing results are shown in Table 1.

As can be seen from Table 1, the obtained results by using Dempster’s combination method are supported by the proposition $\theta_{3}$, while propositions $\theta_{1}$ and $\theta_{2}$ are almost negated. Such a result is considered unreasonable since the evidence $m_{1}$ and $m_{2}$ are assigned only a small amount of their belief value to proposition $\theta_{3}$, but achieved almost the certainty support result. Our proposed method can reasonably assign the conflict and get more acceptable results. Since the maximum belief value is assigned to proposition $\theta_{1}$, the fusion result assigns the maximum belief value to $\theta_{1}$. Two bodies of evidence assign the least belief to proposition $\theta_{3}$, and only a small amount of belief value is assigned to proposition $\theta_{3}$ after fusion, which is consistent with the intuitive analysis.

Suppose that the basic probability distribution of evidence $m_{1}$ changes slightly, and the basic probability distribution after changes is shown as follows:

$$m_{11}:\;m_{1}(\theta_{1})=0.98\;m_{1}(\theta_{2})=0.01\;m_{1}(\theta_{3})=0.01$$

The same method is used to combine the change evidence, and the fusion results are shown in Table 2.

As can be seen from Table 2, although the evidence $m_{1}$ has changed only slightly, the result by using Dempster’s combination method changes a lot. The degree of belief in proposition $\theta_{3}$ has changed from the previous complete support to an almost negative result, which indicates that Dempster’s combination method has poor robustness in the focal element. Meanwhile, the result of using our proposed method was almost unchanged. The slight change in the evidence has little impact on the proposed method, which indicates that our proposed method has good robustness.

**Example** **11.** *Suppose that in the same frame of discernment Θ={A,B,C}, the system has collected the data information from three different types of sensors, and the basic probability distribution of each sensor reading is shown in Table 3.*

(1) The Hellinger distance and sine value of the Pignistic vector angle between the evidence are calculated as follows:

$$\begin{array}{c} Hel(m_{1},m_{2})=0.4531\;Hel(m_{1},m_{3})=0.4590\;Hel(m_{2},m_{3})=0.0173\\ Sin(m_{1},m_{2})=0.5146\;Sin(m_{1},m_{3})=0.5381\;Sin(m_{2},m_{3})=0.0381 \end{array}$$

(2) Calculate the mutual support degree among the evidence, and construct the support degree matrix as follows:

$$SM=\left[\begin{array}{ccc} 1 & 0.2655 & 0.2499\\ 0.2655 & 1 & 0.9453\\ 0.2499 & 0.9453 & 1 \end{array}\right]$$

(3) Calculate the average support degree of each body of evidence as follows:

$$Sup(m_{1})=0.2577\;Sup(m_{2})=0.6054\;Sup(m_{3})=0.5976$$

(4) Compute the credibility degree of each body of evidence as follows:

$$Cred(m_{1})=0.1764\;Cred(m_{2})=0.4145\;Cred(m_{3})=0.4091$$

Then, the weighting factor of each body of evidence is:

$$\omega_{1}=0.1764\;\omega_{2}=0.4145\;\omega_{3}=0.4091$$

(5) According to the weight factor of each body of evidence, the weighted average evidence is calculated as follows:

$$\begin{array}{c} m(A)=0.3059\;m(B)=0.3641\;m(C)=0.0265\;m(\{A,B\})=0.2735\\ m(\{A,C\})=0.0124\;m(\{B,C\})=0.0124\;m(\Theta )=0.0052 \end{array}$$

(6) Combine the weighted average evidence through Dempster’s combination rule twice, and the fusing results are shown in Table 4.

It can be seen from Table 3, for the sensor $m_{1}$, the most supported hypothesis is A while it is B for sensors $m_{2}$ and $m_{3}$. So, the sensor $m_{1}$ is a highly conflicting evidence that has a negative impact on the fusion results. According to the intuitive analysis, we can know that the fusion results will support the hypothesis B to a great extent.

As shown in Table 4, the obtained results by using Martin’s [43] and Jiang’s [60] method are almost identical to those obtained through Dempster’s combination method. They all assign the maximum belief value to hypothesis A, which contradicts the intuitive analysis. In fact, in Martin’s method and Jiang’s method, they consider that three sensors have almost the same reliability since they only use the dissimilarity to evaluate the degree of conflict between the evidence. One single measurement method cannot accurately assess the degree of conflict between the evidence. By contrast, the obtained results by using our proposed method provide a greater belief value to support the hypothesis B, which is consistent with the intuitive analysis. The main reason for this result is that the proposed method takes into account the impact of the dissimilarity and inconsistency between the evidence on conflict in the evaluation of the evidence conflict. The result shows that sensors $m_{2}$ and $m_{3}$ are the most reliable and, therefore, have the most important effect on the fusion results. So, the fusion results are more reasonable.

The above two examples show that when dealing with high conflicting evidence, our proposed method can accurately evaluate the degree of conflict between the evidence, and have high decision-making efficiency and good robustness.

## 7. Experiment Analysis

In order to verify and analyze the effectiveness and rationality of the proposed method, we illustrate through a numerical example and a simplified fault diagnosis problem in this section, a present test and comparative analysis with some of the approaches that are currently available in the literature.

### 7.1. Numerical Example

Assume that there is a multi-sensor target recognition problem, each sensor can display the relevant data of the target type being detected. Suppose there are three types of target $\theta_{1}$, $\theta_{2}$, and $\theta_{3}$, and these target types constitute the frame of discernment Θ. In the same frame of discernment $\Theta =\{\theta_{1},\;\theta_{2},\;\theta_{3}\}$, the system has collected the data information from five different types sensors, and the basic probability distribution of each sensor reading is shown in Table 5.

As can be seen from Table 5, we can note that four sensors $m_{1}$, $m_{2}$, $m_{4}$, and $m_{5}$ assign most of their belief value to target $\theta_{1}$, while sensor $m_{3}$ gives its largest belief value to target $\theta_{2}$. Obviously, $m_{3}$ is an abnormal evidence. Therefore, $m_{3}$ highly conflicts with other evidence. The fusion results obtained by different combination methods are shown in Table 6.

### 7.2. Discussion

**Figure 5:**
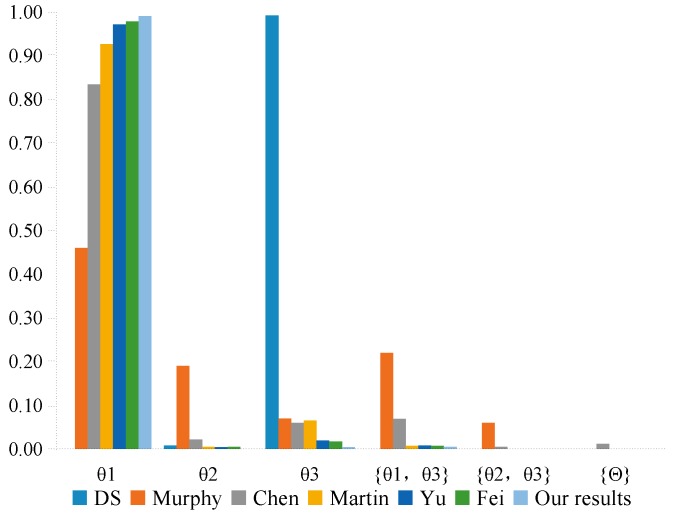
The comparison of the fusion results in different methods.

**Figure 6:**
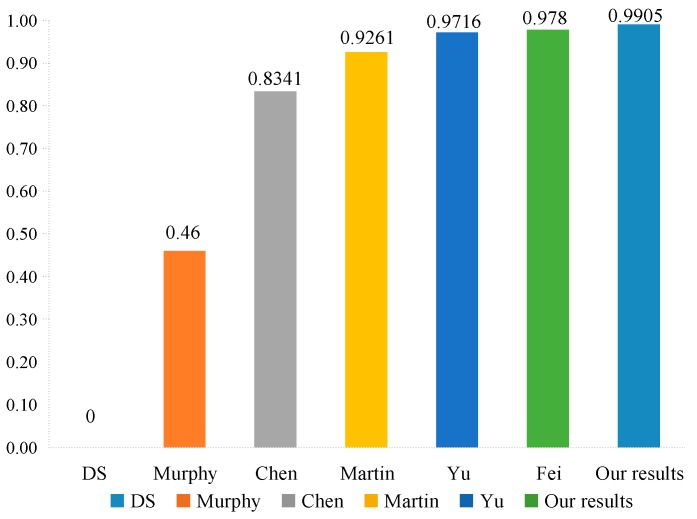
The comparison of the belief value allocation for target $\theta_{1}$ in different methods.

As shown in Table 6, according to the combination of all sensors using Dempster’s combination method, $\theta_{2}$ is strongly supported as the target while the belief value of the target $\theta_{1}$ is 0. Such a result is considered unreasonable since the majority of sensors assign most of their belief value to $\theta_{1}$ and only sensor $m_{3}$ assigns a large of belief value to $\theta_{2}$, which is inconsistent with the intuitive analysis. In the case of high conflict, using Dempster’s combination method will achieve the wrong decision result. However, in Murphy’s [41] method, Martin’s [43] method, Chen’s [49] method, Yu’s [51] method, Fei’s [61] method, as well as in our proposed method, one can obtain reasonable results and recognize the target $\theta_{1}$, as shown in Figure 5. Murphy’s method only simplifies the averages of each evidence without considering their credibility, and the obtained result has the least support for the target $\theta_{1}$ compared with other methods. Such a result is not conducive to decision-making. In fact, each body of evidence may not have the same importance, so we need to allocate the weight reasonably based on the reliability of each body of evidence. Martin’s method, Chen’s method, and Yu’s method are used as the evidence distance to measure the dissimilarity between the evidence so as to characterize the degree of conflict between the evidence. Fei’s method uses a new divergence to measure the difference between different basic probability assignments, and utilizes the divergence to calculate the similarity between the evidence. These methods can obtain reasonable results, but they have a slow speed in convergence. It is shown that only use a single measurement method cannot effectively evaluate the degree of conflict between the evidence. In this paper, our proposed method comprehensively considers the impact of the dissimilarity and inconsistency between the evidence on conflicts, and the combination results support target $\theta_{1}$ more than those obtained by other approaches, as shown in Figure 6. It illustrates that our proposed method are more efficient and convergent in dealing with conflict evidence. In our method, the evidence that has high conflicts with other evidence will be assigned low credibility to reduce the impact of unreliable evidence on the fusion results, while the evidence with non-conflict obtained higher credibility, making them play a more important role in the fusion process. Such credibility allocation makes the fusion results more accurate and reasonable.

### 7.3. Fault Diagnosis Problem

In industrial production, mechanical systems will have various types of fault. In order to quickly repair the machine fault, we need to accurately determine the type of machine fault. Suppose that there are three different types of faults in some types of machine systems, which constitutes the frame of discernment $\Theta =\{F_{1},\;F_{2},\;F_{3}\}$ in the fault diagnosis problem. In this paper, the fault diagnosis system used three sensors, $S=\{S_{1},\;S_{2},\;S_{3}\}$, which distributed in different locations to collect the faulty information. The basic probability assignment function is applied to each model to collect the fault information by sensors. The basic probability assignment of each sensor data is obtained as shown in Table 7. $m_{1}$, $m_{2}$, and $m_{3}$ represent the basic probability assignments reported from the three sensors $S_{1}$, $S_{2}$, and $S_{3}$, respectively.

In Table 7, we can note that $m_{2}$ strongly supports the fault type $F_{2}$, while $m_{1}$ and $m_{3}$ strongly support the fault type $F_{1}$. Therefore, there is a highly conflicting between the evidence $m_{2}$ and the other two bodies of evidence. Using our proposed method to deal with the conflict evidence, the calculation process is as follows:

(1) The Hellinger distance and sine value of the Pignistic vector angle between the evidence are calculated as follows:

$$\begin{array}{c} Hel(m_{1},m_{2})=0.5761\;Hel(m_{1},m_{3})=0.1030\;Hel(m_{2},m_{3})=0.5995\\ Sin(m_{1},m_{2})=0.9146\;Sin(m_{1},m_{3})=0.0616\;Sin(m_{2},m_{3})=0.9405 \end{array}$$

(2) Calculate the mutual support degree between the evidence, and construct the support degree matrix as follows:

$$SM=\left[\begin{array}{ccc} 1 & 0.0362 & 0.8417\\ 0.0362 & 1 & 0.0238\\ 0.8417 & 0.0238 & 1 \end{array}\right]$$

(3) Calculate the average support degree of each body of evidence as follows:

$$Sup(m_{1})=0.4390\;Sup(m_{2})=0.03\;Sup(m_{3})=0.4328$$

(4) Compute the credibility degree of each body of evidence as follows:

$$Cred(m_{1})=0.4868\;Cred(m_{2})=0.0333\;Cred(m_{3})=0.4799$$

(5) In fault diagnosis applications, we also need to calculate the static reliability of the evidence. The parameters related to static reliability are sufficiency index μ(m) and importance index υ(m). The sufficiency index μ(m) and importance index υ(m) parameters of the evidence in the application of fault diagnosis obtained from the literature [62] are shown in Table 8.

The static reliability of the evidence can be calculated by using these parameters. The calculation formula is as follows:

(21) $$\begin{array}{c} W{\left(SR\right)}_{i}=\mu (m_{i})\times \upsilon (m_{i})\;i\in [1,\;n]\\ W{\left(SR\right)}_{1}=1.0000\;W{\left(SR\right)}_{2}=0.2040\;W{\left(SR\right)}_{3}=1.0000 \end{array}$$

(6) According to the static reliability and the credibility degree of each body of evidence, the final weight of each evidence is calculated as follows:

$$\begin{array}{c} \omega_{1}=W{\left(SR\right)}_{1}\times Cred(m_{1})=0.4868\\ \omega_{2}=W{\left(SR\right)}_{2}\times Cred(m_{2})=0.0068\\ \omega_{3}=W{\left(SR\right)}_{3}\times Cred(m_{3})=0.4799 \end{array}$$

(7) Normalize the final weight of the evidence and obtain the weight as shown below:

$${\tilde{\omega }}_{1}=0.5000\;{\tilde{\omega }}_{2}=0.0070\;{\tilde{\omega }}_{3}=0.4930$$

(8) According to the weight factor of each body of evidence, the weighted average evidence is calculated as follows:

$$m(F_{1})=0.6455\;m(F_{2})=0.1049\;m(\{F_{2},\;F_{3}\})=0.0996\;m(\{F_{1},\;F_{2},\;F_{3}\})=0.1500$$

(9) Combine the weighted average evidence through Dempster’s combination rule twice, and the fusing results are shown in Table 9.

In this paper, for comparative analysis, we use different methods for fusion. The fusion results obtained through different combination methods are shown in Table 9.

### 7.4. Discussion

**Figure 7:**
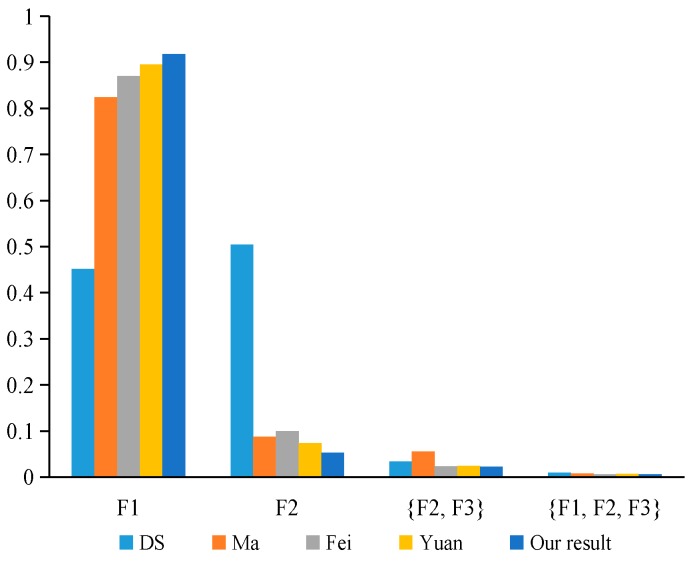
The comparison of the fusion results in different methods.

**Figure 8:**
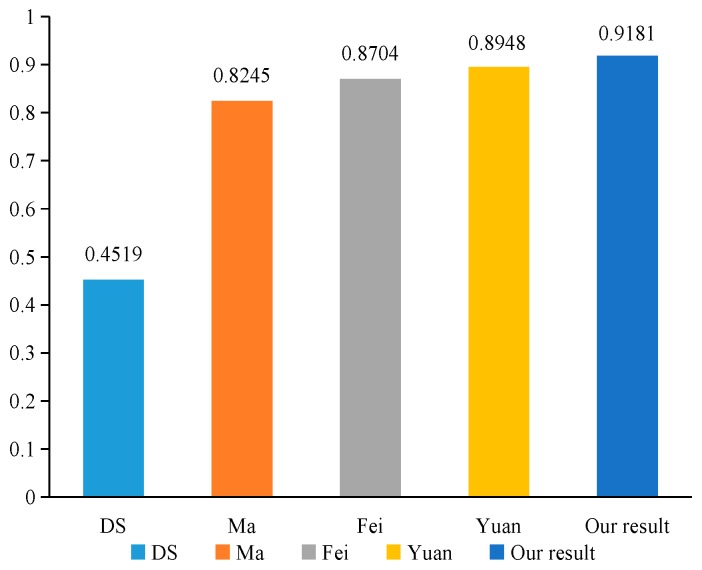
The comparison of the belief value for fault type $F_{1}$ in different methods.

As shown in Table 9, Dempster’s combination method cannot handle the conflicting evidence very well, and lead to the wrong fusion result that the fault type $F_{2}$ is supported. Such a result is considered unreasonable since the majority of sensors assign most of their belief value to $F_{1}$ and only sensor $m_{2}$ distributes a large belief value to $F_{2}$. However, Ma’s [62] method, Fei’s [61] method, Yuan’s [9] method, and our proposed method can effectively diagnose the fault type as $F_{1}$, as shown in Figure 7. When there is highly conflicting evidence, our proposed method and the above methods that are mentioned in this article can effectively deal with the conflict evidence. Furthermore, we note that in our proposed method, the combination results support the fault type $F_{1}$ more than those obtained by other approaches, as shown in Figure 8. This is due to the fact that Ma’s method only considers the impact of the dissimilarity between the evidence on conflicts, Fei’s method only uses the similarity between the evidence to describe the degree of conflict between the evidence, and Yuan’s method only considers the impact of uncertainty and dissimilarity for the evidence on conflicts. These methods take into account only a single aspect that affects the conflict of evidence, and do not reflect the real-world situation. Therefore, they cannot accurately measure the degree of conflict between the evidence. However, our proposed method comprehensively considers the impact of the dissimilarity and inconsistency between the evidence on conflicts, can effectively evaluate the degree of conflict between the evidence, and can reasonably allocate the credibility of each evidence. Hence, our proposed method has the best performance with convergence and higher precision. Experimental results show that our proposed method is more reasonable and effective in dealing with highly conflicting evidence.

## 8. Conclusions

DS evidence theory is an effective tool for multi-sensor data fusion, which is widely applied in the field of information fusion. However, Dempster’s combination method cannot effectively manage conflicts between different information sources. When fusing the high conflicting evidence with Dempster’s combination rule, the anti-intuitive results will be obtained. How to deal with the high conflicting evidence effectively remains to be solved. In this paper, by considering both of the effects of the evidence distance and evidence angle on conflict, a novel method for conflicting evidence fusion based on the dissimilarity and inconsistency between the evidence is proposed. We use the Hellinger distance to measure the distance between the evidence, and then the dissimilarity between the evidence is described by the evidence distance. The sine value of the Pignistic vector angle is utilized to calculate the magnitude of the angle between the evidence, and the inconsistency between the evidence is represented by the evidence angle. On the basis of the dissimilarity and inconsistency between the evidence, a new method for the mutual support degree between the evidence is presented to characterize the degree of conflict between the evidence. Several examples are given to illustrate the applicability and effectiveness of the proposed method. Compared with other methods, the proposed method can accurately evaluate the degree of conflict between the evidence, obtain more reasonable fusion results, and have a faster speed in convergence, which can be applied to many practical problems.

Considering the proposed method in this work can effectively deal with the conflicting evidence and overcome the problems existing in traditional methods, in future work, we intend to generalize this method to other uncertainty theories, such as fuzzy set theory and imprecise probabilities. In addition, we will also research the total uncertainty of evidence directly in the frame of discernment by using the idea of this method.

## Figures and Tables

**Table 1:** Combination results of the two bodies of evidence $m_{1}$ and $m_{2}$.

	$\theta_{1}$	$\theta_{2}$	$\theta_{3}$
DS	0	0	1
Our result	0.5438	0.4495	0.0067

**Table 2:** Combination results of the modified evidence $m_{1}$ and evidence $m_{2}$.

	$\theta_{1}$	$\theta_{2}$	$\theta_{3}$
DS	0	0.9	0.1
Our result	0.5334	0.4599	0.0067

**Table 3:** Basic probability distribution of multi-sensor data.

	A	B	C	{A, B}	{A,C}	{B,C}	{Θ}
$m_{1}$	0.38	0.15	0.15	0.15	0.07	0.07	0.03
$m_{2}$	0.30	0.40	0	0.30	0	0	0
$m_{3}$	0.28	0.42	0	0.30	0	0	0

**Table 4:** Combination results of the evidence $m_{1}$, $m_{2}$ and $m_{3}$.

	A	B	C	{A, B}	{A,C}	{B,C}	{Θ}	Decision
DS	0.502	0.458	0	0.040	0	0	0	A
Martin	0.491	0.462	0	0.047	0	0	0	A
Jiang	0.452	0.438	0.005	0.092	0.002	0.002	0.009	A
Our results	0.4044	0.5494	0.0004	0.0458	0	0	0	B

**Table 5:** Basic probability distribution of multi-sensor data.

	$\theta_{1}$	$\theta_{2}$	$\theta_{3}$	$\left\{\theta_{1},\theta_{3}\right\}$	$\left\{\theta_{2},\theta_{3}\right\}$
$m_{1}$	0.65	0.05	0.25	0.05	0
$m_{2}$	0.55	0.10	0	0.35	0
$m_{3}$	0	0.60	0.10	0	0.30
$m_{4}$	0.55	0.10	0	0.35	0
$m_{5}$	0.55	0.10	0	0.35	0

**Table 6:** The comparison of the fusion results with different methods.

	$\theta_{1}$	$\theta_{2}$	$\theta_{3}$	$\left\{\theta_{1},\theta_{3}\right\}$	$\left\{\theta_{2},\theta_{3}\right\}$	{Θ}
DS	0	0.008	0.992	0	0	0
Murphy	0.460	0.190	0.070	0.220	0.060	0
Martin	0.9261	0.0013	0.0652	0.0074	0	0
Chen	0.8341	0.0223	0.0604	0.0692	0.0017	0.0123
Yu	0.9716	0.0004	0.0198	0.0082	0	0
Fei	0.9780	0.0010	0.0173	0.0037	0	0
Our results	0.9905	0	0.0044	0.0051	0	0

**Table 7:** The basic probability distribution of sensor data in fault diagnosis problem.

	$F_{1}$	$F_{2}$	$\left\{F_{2},\;F_{3}\right\}$	$\left\{F_{1},\;F_{2},\;F_{3}\right\}$
$m_{1}$	0.60	0.10	0.10	0.20
$m_{2}$	0.05	0.80	0.05	0.10
$m_{3}$	0.70	0.10	0.10	0.10

**Table 8:** Parameters in the fault diagnosis application.

Evidence	$m_{1}$	$m_{2}$	$m_{3}$
Sufficiency index μ(m)	1.00	0.60	1.00
Importance index υ(m)	1.00	0.34	1.00

**Table 9:** Fusion results of different combination methods

	$F_{1}$	$F_{2}$	$\left\{F_{2},\;F_{3}\right\}$	$\left\{F_{1},\;F_{2},\;F_{3}\right\}$
DS	0.4519	0.5048	0.0336	0.0096
Ma	0.8245	0.0877	0.0553	0.0080
Fei	0.8704	0.0999	0.0232	0.0065
Yuan	0.8948	0.0739	0.0241	0.0072
Our result	0.9181	0.0533	0.0224	0.0062

## References

[1] Lin G., Liang J., Qian Y. (2015). An information fusion approach by combining multi-granulation rough sets and evidence theory. Inf. Sci..

[2] Liu H.C., Liu L., Lin Q.L. (2013). Fuzzy failure mode and effects analysis using fuzzy evidential reasoning and belief rule based methodology. IEEE Trans. Reliab..

[3] Xiao F.Y. (2018). An improved method for combining conflicting evidences based on the similarity measure and belief function entropy. Int. J. Fuzzy Syst..

[4] Zheng H., Deng Y. (2017). Evaluation method based on fuzzy relations between Dempster-Shafer belief structure. Int. J. Intell. Syst..

[5] Xiao F.Y. (2017). A novel evidence theory and fuzzy preference approach-based multi-sensor data fusion technique for fault diagnosis. Sensors.

[6] Luo H., Yang S., Hu X. (2012). Agent oriented intelligent fault diagnosis system using evidence theory. Exp. Syst. Appl..

[7] Du Y., Lu X., Su X., Hu Y., Deng Y. (2016). New failure mode and effects analysis: An evidential downscaling method. Qual. Reliab. Eng. Int..

[8] Jiang W., Xie C., Zhuang M., Tang Y. (2017). Failure mode and effects analysis based on a novel fuzzy evidential method. Appl. Soft Comput..

[9] Yuan K., Xiao F.Y., Fei L.G., Kang B.Y., Deng Y. (2016). Modeling sensor reliability in fault diagnosis based on evidence theory. Sensors.

[10] Cheng G., Chen X.H., Shan X.L., Liu H.G., Zhou C.F. (2016). A new method of gear fault diagnosis in strong noise based on multi-sensor information fusion. J. Vib. Control.

[11] An J.Y., Hu M., Fu L., Zhan J.W. (2019). A novel fuzzy approach for combining uncertain conflict evidences in the Dempster-Shafer theory. IEEE Access.

[12] Yager R.R., Alajlan N. (2015). Dempster-Shafer belief structures for decision making under uncertainty. Knowl. Based Syst..

[13] Chao F., Yang S.L. (2014). Conjunctive combination of belief functions from dependent sources using positive and negative weight functions. Expert Syst. Appl..

[14] Fu C., Xu D.L. (2016). Determining attribute weights to improve solution reliability and its application to selecting leading industries. Ann. Oper. Res..

[15] Deng X.Y., Jiang W. (2017). An evidential axiomatic design approach for decision making using the evaluation of belief structure satisfaction to uncertain target values. Int. J. Intell. Syst..

[16] Xiao F.Y. (2019). EFMCDM: Evidential fuzzy multicriteria decision making based on belief entropy. IEEE Trans. Fuzzy Syst..

[17] Kabir G., Tesfamariam S., Francisque A., Sadiq R. (2015). Evaluating risk of water mains failure using a Bayesian belief network model. Eur. J. Oper. Res..

[18] Dutta P. (2015). Uncertainty modeling in risk assessment based on Dempster-Shafer theory of evidence with generalized fuzzy focal elements. Fuzzy Inf. Eng..

[19] Deng X.Y., Jiang W. (2017). Fuzzy risk evaluation in failure mode and effects analysis using a D numbers based multi-sensor information fusion method. Sensors.

[20] Zheng X., Deng Y. (2017). Dependence assessment in human reliability analysis based on evidence credibility decay model and IOWA operator. Ann. Nucl. Energy.

[21] Zhang L., Ding L., Wu X., Skibniewski M.L. (2017). An improved Dempster-Shafer approach to construction safety risk perception. Knowl. Based Syst..

[22] Fei L.G., Xia J., Feng Y.Q., Liu L.N. (2019). An ELECTRE-based multiple criteria decision making method for supplier selection using Dempster-Shafer theory. IEEE Access.

[23] Fei L.G., Deng Y., Hu Y. (2019). DS-VIKOR: A new multi-criteria decision-making method for supplier selection. Int. J. Fuzzy Syst..

[24] Liu Z.G., Pan Q., Dezert J., Martin A. (2016). Adaptive imputation of missing values for incomplete pattern classification. Pattern Recognit..

[25] Dong G., Kuang G. (2015). Target recognition via information aggregation through Dempster-Shafer’s evidence theory. IEEE Geosci. Remote Sens. Lett..

[26] Denoeux T. (1995). A k-nearest neighbor classification rule based on Dempster-Shafer theory. IEEE Trans. Syst. Man Cybernet.

[27] Ma J., Liu W., Miller P., Zhou H. (2016). An evidential fusion approach for gender profiling. Inf. Sci..

[28] Dempster A.P. (1967). Upper and lower probabilities induced by a multivalued mapping. Ann. Math. Stat..

[29] Shafer G. (1976). A Mathematical Theory of Evidence.

[30] Zhang Z.J., Liu T.H., Dong C., Zhang W.Y. (2014). Novel algorithm for identifying and fusing conflicting data in wireless sensor networks. Sensors.

[31] Zadeh L.A. (1986). A simple view of the Dempster–Shafer theory of evidence and its implication for the rule of combination. AI Mag..

[32] Deng Y. (2016). Deng entropy. Chaos Solitons Fractals.

[33] Tao R., Xiao F.Y. (2019). Combine conflicting evidence based on the belief entropy and IOWA operator. IEEE Access.

[34] Deng X.Y., Xiao F.Y., Deng Y. (2017). An improved distance-based total uncertainty measure in belief function theory. App. Int..

[35] Song Y.T., Deng Y. (2019). A new method to measure the divergence in evidential sensor data fusion. Int. J. Distrib. Sens. Netw..

[36] Jiang W., Zhan J. (2017). A modified combination rule in generalized evidence theory. Appl. Intell..

[37] Denoeux T. (2008). Conjunctive and disjunctive combination of belief functions induced by nondistinct bodies of evidence. Artif. Intell..

[38] Lefevre E., Elouedi Z. (2013). How to preserve the conflict as an alarm in the combination of belief functions?. Decis. Support Syst..

[39] Xiao F.Y., Qin B.W. (2018). A weighted combination method for conflicting evidence in Multi-Sensor data fusion. Sensors.

[40] Pan L.P., Deng Y. (2018). A new belief entropy to measure uncertainty of basic probability assignments based on belief function and plausibility function. Entropy.

[41] Murphy C.K. (2000). Combining belief functions when evidence conflicts. Decis. Support Syst..

[42] Jousselme A.L., Grenier D., Bosse E. (2001). A new distance between two bodies of evidence. Inf. Fusion.

[43] Martin A., Jousselme A.L., Osswald C. Conflict measure for the discounting operation on belief functions. Proceedings of the 2008 11th International Conference on Information Fusion Conference.

[44] Deng Y., Shi W.K., Zhu Z.F., Liu Q. (2004). Combining belief functions based on distance of evidence. Decis. Support Syst..

[45] Han D.Q., Deng Y., Han C.Z., Hou Z.Q. (2011). Weighted evidence combination based on distance of evidence and uncertainty measure. J. Infrared Millim. Waves.

[46] Smets P., Kennes R. (1994). The transferable belief model. Artif. Intell..

[47] Liu W. (2006). Analyzing the degree of conflict among belief functions. Artif. Intell..

[48] Zhang Y., Xu T., Yang G.Q. (2014). A reliability analysis of airport noise monitoring data based on evidence theory. J. Comput..

[49] Chen Y.E., Xia X.Z., Ge S. (2014). An approach to conflict evidence combination based on two criteria optimization. J. Comput. Inf. Syst..

[50] Xiao F.Y. (2019). Multi-sensor data fusion based on the belief divergence measure of evidences and the belief entropy. Inf. Fusion.

[51] Yu C., Yang J.H., Yang D.B., Ma X.H., Min H.C. (2015). An improved conflicting evidence combination approach based on a new supporting probability distance. Expert Syst. Appl..

[52] Yang Y., Han D.Q. (2016). A new distance-based total uncertainty measure in the theory of belief functions. Knowl. Based Syst..

[53] Burger T. (2016). Geometric views on conflicting mass functions: From distance to angles. Int. J. Approx. Reason..

[54] Csiszar I. (1967). Information type measures of difference of probability distributions and indirect observations. Studia Sci. Math. Hung..

[55] Ali M.S., Silvey S.D. (1966). A general class of coefficients of divergence of one distribution from another. J. R. Stat. Soc..

[56] Wang S.F. (2008). Research of Cooperation in Ad Hoc Networks Based on Hellinger Distance. Master’s Thesis.

[57] Lee C.H., Shind G. (1999). Using Hellinger distance in a nearest neighbour classifier for relational databases. Knowl. Based Syst..

[58] Fisch D., Janicke M., Sick B. Quantitative Emergence: A refined approach based on divergence measures. Proceedings of the 4th IEEE International Conference on Self-Adaptive and Self-Organizing Systems.

[59] Jin Z., Xin G., Liu H.Q. (2018). A new conflict evidence decision method and its application. J. Beijing Univ. Aeronaut. Astronaut..

[60] Jiang W., Zhang A., Yang Q. A new method to determine evidence discounting coefficients. Proceedings of the 4th International Conference on Intelligent Computing.

[61] Fei L.G., Deng Y. (2019). A new divergence measure for basic probability assignment and its application in extremely uncertain environments. Int. J. Intell. Syst..

[62] Ma M.M., An J.Y. (2015). Combination of evidence with different weighting factors: A novel probabilistic based dissimilarity measure approach. J. Sens..

